# Effect of buoyancy loads on the tsunami fragility of existing reinforced concrete frames including consideration of blow-out slabs

**DOI:** 10.1038/s41598-023-36237-7

**Published:** 2023-06-02

**Authors:** Marta Del Zoppo, Tiziana Rossetto, Marco Di Ludovico, Andrea Prota

**Affiliations:** 1 EPICentre, Department of Civil, Environmental and Geomatic Engineering, University College London, London, UK; 2 Department of Structures for Engineering and Architecture, University of Naples Federico II, Naples, Italy

**Keywords:** Engineering, Civil engineering

## Abstract

Currently available performance-based methodologies for assessing the fragility of structures subjected to tsunami neglect the effects of tsunami-induced vertical loads due to internal buoyancy. This paper adopts a generalized methodology for the performance assessment of structures that integrates the effects of buoyancy loads on interior slabs during a tsunami inundation. The methodology is applied in the fragility assessment of three case-study frames (low, mid and high-rise), representative of existing masonry-infilled reinforced concrete (RC) buildings typical of Mediterranean region. The paper shows the effect of modelling buoyancy loads on damage evolution and fragility curves associated with different structural damage mechanisms for existing RC frames with breakaway infill walls including consideration of blow-out slabs. The outcomes attest that buoyancy loads affect the damage assessment of buildings during a tsunami, especially in the case of mid and high-rise structures with blow-out slabs. The rate of occurrence of slabs uplift failure increases with the number of stories of the building, indicating the need to account for such damage mechanism when assessing the performance of structures. It is also found that buoyancy loads slightly affect the fragility curves associated to other structural damage mechanisms for existing RC buildings commonly monitored for fragility assessment.

## Introduction

Far-fault tsunami events have a low frequency but can induce high human and economic losses on coastal communities^1–3^. The fragility assessment of existing assets against tsunami-induced loads is fundamental for estimating the resilience of the built environment to such hazard and to inform disaster risk management. A comprehensive description of tsunami-induced loads and effects on structures has been reported in the recent ASCE 7-22 Standard^4^ for the design of tsunami vertical shelters. However, the Standard does not explicitly state how to apply tsunami-induced loads on buildings in a non-linear structural analysis approach for the fragility assessment of structures. Recent studies have proposed numerical tools for the performance or fragility assessment of structures during a tsunami inundation under tsunami-induced drag loads^5–9^. Only a very few studies in tsunami engineering have considered uplift pressure due to buoyancy in their structural analyses. Tokimatsu et al.^10^ and Chaudhary et al.^11^, among few others, have looked at buoyancy loads on structures with main focus on buoyancy acting at foundation level to assess the resistance against global overturning of buildings. In buildings with breakaway infill walls (i.e., walls that fail out of their plane during a tsunami inundation), the magnitude of buoyancy loads acting at foundation level after the failure of exterior cladding is relatively low due to the limited enclosed space inside the structure, and global overturning is unlikely to happen^1^. However, internal buoyancy induces uplift loads on elevated slabs due to enclosed spaces inside the structure, air trapped below floors (i.e., air pockets) and submerged structural members that have been largely ignored in tsunami fragility and vulnerability analysis performed so far. Tsunami buoyancy induced loads may represent a source of damage for elevated slabs of structures with breakaway walls in coastal area, causing the uplift failure of blow-out slabs^1,12^. This damage mechanism has never been included in past analytical fragility and vulnerability studies. Buoyancy can also cause a reduction of axial load in vertical members, with a consequent reduction of flexural and shear capacity that can lead to premature local and global failure mechanisms for the building^13^. Yet, the effect of tsunami-induced vertical loads on elevated slabs due to internal buoyancy has not been properly addressed in the literature regarding the structural assessment of buildings subjected to tsunami. Few experimental tests in hydraulic flumes have been performed to assess uplift forces on slabs and bridge decks induced by buoyancy^14–16^. These studies represent a valuable simulation of the effect of waves on elevated slabs in terms of loads and pressure. However, such findings have not yet been integrated into tsunami structural analysis methodologies, and empirically validated load equations are lacking.

This paper adopts a generalized analysis methodology, called Variable Depth Pushover for Breakaway Infilled frames (VDPO-BI), for including buoyancy and lateral loads in the tsunami performance assessment of structures. The VDPO-BI is adopted to investigate the significance of the inclusion of buoyancy loads and blow-out slabs on the tsunami fragility of reinforced concrete (RC) frames associated to selected structural damage mechanisms. Three case-study masonry infilled RC frames typical of the Mediterranean region with different heights are adopted to develop fragility curves at single building level, pointing out the correlation between buildings height and effects of buoyancy loads on RC structures. Structural damage levels are defined based on the HAZUS Tsunami Technical Manual Guidance^17^, and the effects of buoyancy and blow-out slabs on the fragility curves for the case-study frames are presented and discussed.

## Methods

The performance of existing RC buildings to tsunami-induced lateral and vertical loads is herein investigated by means of refined FEM analyses performed through the generalised VDPO-BI. In the following, the models selected to simulate lateral and vertical tsunami on-shore flows on structures are first illustrated; then, the VDPO-BI procedure is briefly presented to assess the effect of buoyancy loads on the fragility of RC frames with breakaway infill walls and blow-out slabs.

### Tsunami-induced loads on structures

Tsunami-induced lateral loads on structures mainly consist of unbalanced hydrostatic pressure, hydrodynamic or drag pressure, bore forces and debris impact loads, while vertical loads are mainly related to hydrostatic buoyancy and hydrodynamic surge^4^. Many of these loads are impulsive and highly transient in nature, and hence are neglected in the VDPO-BI. Indeed, in the VDPO-BI analysis the tsunami is simulated as a steady state flow (hydrostatic and hydrodynamic loads), and the initial bore phase is ignored. Impact loads are extremely short duration impulsive loads and cannot be represented by pushover analysis. In the context of ASCE 7, the hydrodynamic and hydrostatic loads need not be combined with the debris impact loads because of the low probability of simultaneous occurrence of the maximum of each type of loading^18^. Soil erosion and scour induced by the tsunami flow are also neglected in this study. Lateral and buoyancy induced loads on structures considered in the VDPO-BI are further discussed in next sections.

### Lateral loads

Lateral loads in the form of unbalanced hydrostatic and hydrodynamic pressures are considered acting on vertical components of a structure (i.e., infill walls and columns). Although in the VDPO-BI any assumption can be made as to the lateral load formulation and pressure distribution applied to the structure, in this paper the lateral load equations proposed by Foster et al.^19^ are used. These equations are chosen as they have been empirically validated with recent experimental investigations performed in hydraulic flumes. According to Foster et al., the overall lateral tsunami force has different formulations as a function of the flow regime, which is described by the Froude number (i.e., $Fr= u/\sqrt{{gH}_{w}}$, with *u* the flow velocity) as reported in the following:

1 $ \begin{gathered}   F_{t} /b = 0.5C_{D} \rho u^{2} H_{w} \quad \quad \,subcritical\,regime\,\,if\,Fr < Fr\_{\text{c}} \hfill \\   F_{t} /b = \lambda _{s} \rho g^{{1/3}} u^{{4/3}} H_{w} ^{{4/3}} \quad chocked\,regime\,\,if\,Fr \ge Fr\_{\text{c}} \hfill \\  \end{gathered}  $

where *F*_*t*_*/b* is tsunami force per unit width (i.e., *b* is the impact surface width), *ρ* is the sea water density (1.2 t/m^3^ in order to account for suspended sediment), *g* is the gravitational acceleration, *H*_*w*_ is the tsunami inundation depth, *C*_*D*_ is the drag coefficient for the building and *λs* is the leading coefficient for steady flows. The leading coefficient is computed as a function of the blocking ratio, *B/w* (with *B* the building width, *w* the width of the flume), as follows:

2 $${\lambda }_{s}=0.73+1.2\left(B/w\right)+1.1{\left(B/w\right)}^{2}$$

The blocking ratio is representative of the density of the urban context that affects the flow properties. In a subcritical regime, the Foster et al. equation represents the drag load component. Conversely, in a chocked regime, the overall tsunami force includes both unbalanced hydrostatic and drag loads. Hence, drag loads in the chocked regime are herein computed as the difference between the tsunami force from Eq. (1) and the hydrostatic load, computed according to the ASCE 7. Drag loads are applied on the building assuming a uniform pressure distribution, as prescribed by the ASCE 7. Unbalanced hydrostatic loads acting on seaward structural and non-structural members before the failure of breakaway infill walls are applied with a triangular pressure distribution.

In the VDPO-BI, a constant relation between flow depth and flow velocity is assumed during the analysis by assuming a constant Froude number. This means that flow velocity increases with the inundation depth during the analysis.

### Buoyancy-induced loads

Buoyancy loads on structures are related to the volume of water displaced during the tsunami inundation, according to Archimedes’ principle. Watertight structures or structures with small opening ratios develop large hydrostatic buoyancy loads below the grade of the building due to the enclosed space inside the structure, leading to global overturning mechanisms. Instead, in buildings with breakaway infill walls, buoyancy-induced vertical loads act mainly on interior elevated slabs after the failure of breakaway cladding. Empirically validated models to compute tsunami buoyancy-induced vertical loads on structural components are currently missing. Hence, in this study, the guidance provided in the ASCE 7-22 provisions and by Robertson^18^ are followed. The ASCE 7-22 clearly states that buoyancy includes the effect of air trapped below floors, including integral structural slabs. In the Commentary to the standard ASCE 7-22, is also clarified that the displaced volume of water that contributes to buoyancy should include any structural components, enclosed spaces, floor soffits, and integrated structural slabs where air may be entrapped by beams. However, the standard does not provide explicit equations for each load component.

**Figure 1:**
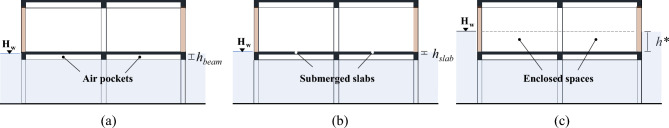
Buoyancy-induced uplift loads on elevated slabs: air pockets (**a**), submerged slabs (**b**) and enclosed spaces (**c**).

Hydrostatic buoyancy-induced loads during tsunami inflow are composed of several components, as shown in Fig. 1 and summarized here:

- Buoyancy due to air pockets: air pockets trapped below elevated slabs, for instance air trapped between consecutive beams and slab soffit (Fig. 1a), induce a maximum uplift pressure on interior slabs computed as:
  3 $$ p_{ap} = \rho gh_{beam} $$

where *h*_*beam*_ is the net height of the beams with respect to the slab, *ρ* is the sea water density,* g* is the gravitational acceleration. This equation provides a conservative estimation of the volume of trapped air neglecting the air compressibility and the possibility of air driven out by water turbulence^18^.

- Buoyancy due to submerged slabs: a reduction of weight due to buoyancy is expected in submerged slabs during the inflow. This uplift pressure due to fully submerged slabs can be computed as function of the volume of water displaced by the slab (height of the slab* h*_*slab*_, see Fig. 1b):
  4 $$ p_{ap} = \rho gh_{slab} $$
  where h* is the height of the enclosed volume, computed as the net distance between the top of the slab and the tsunami inundation depth. This load component strictly depends on the out-of-plane capacity of exterior walls in perimetral frames. Indeed, after the failure of exterior walls, the water is allowed into the building relieving this buoyancy effect.
- Buoyancy due to enclosed spaces: before the exterior walls (or windows) break away, enclosed spaces below the inundation depth level induce uplift pressure on interior slabs (Fig. 1c). This uplift pressure can be computed as follows:
  5 $$ p_{b} = \rho gh^{*} $$
  where h* is the height of the enclosed volume, computed as the net distance between the top of the slab and the tsunami inundation depth. This load component strictly depends on the out-of-plane capacity of exterior walls in perimetral frames. Indeed, after the failure of exterior walls, the water is allowed into the building relieving this buoyancy effect.

It should be noted that tsunami buoyancy-induced uplift loads are only a function of the tsunami inundation depth and, differently from lateral loads, do not depend on other tsunami parameters such as the flow velocity (and therefore Froude number).

### VDPO-BI analysis

**Figure 2:**
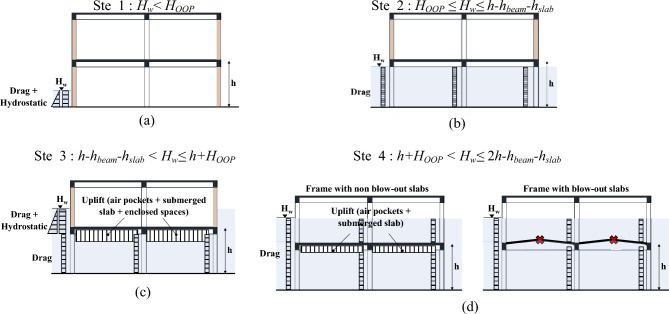
Steps of the generalized VDPO-BI analysis including blow-out slabs.

The generalized VDPO-BI analysis simulates the actual distribution of tsunami-induced loads on structural and non-structural components during the inflow inundation^8^. In the analysis, the tsunami inundation depth, *H*_*w*_, at the site of the structure is monotonically increased up to failure. At each step of inundation depth, the procedure explicitly integrates the tsunami-induced lateral and buoyancy loads acting simultaneously on the structure into the nonlinear static incremental analysis. Hydrodynamic tsunami loads on structures are hence simulated through a static analysis procedure^6^. Some steps of the VDPO-BI including blow-out slabs are depicted in Fig. 2. It should be noted that the analysis methodology involves only the superstructure, and it assumes the structure to be rigidly fixed to the ground.

In the case of frames with breakaway infill walls, the distribution of tsunami-induced loads on structural components strongly depends on the integrity of the infill panels. The inundation depth causing the out-of-plane failure of the walls in the plane normal to the tsunami flow direction is here termed *H*_*OOP*_. Before the out-of-plane failure of the infill walls is reached (i.e., *H*_*w*_ < *H*_*OOP*_), lateral loads are assumed to act solely on the exterior of the building, and hence, infill walls and seaward columns only are directly loaded by tsunami-induced lateral loads, see Fig. 2a. In this phase, uplift loads caused by buoyancy develop below the grade of the building and act on the foundation system. Such uplift loads may cause the global overturning of the building, especially when shallow foundations are used. However, given the low capacity of breakaway infill walls, buoyancy loads on foundation are relatively low in this phase and are therefore ignored in the analysis. Before the failure of exterior infill walls, unbalanced hydrostatic loads locally act on seaward structural and non-structural components. Once the exterior infill walls fail, the water is free to enter the ground floor, inducing lateral drag loads on both exterior and interior columns, see Fig. 2b, while hydrostatic loads are globally balanced. When the inundation depth overcomes the soffit of the first story beams, internal buoyancy due to air pockets and submerged slabs generates uplift loads on slabs (computed as in Eqs. 3 and 4), see Fig. 2c. These act on the structure simultaneously to the lateral loads. For water depths exceeding the first story slab, additional uplift loads caused by buoyancy due to submerged slabs and enclosed spaces will act on the first story slab (Eq. 5), as reported Fig. 2c. This uplift load component increases with the inundation depth until the failure of the second story infill walls is reached. Then, uplift loads due to enclosed spaces are relieved, see Fig. 2d. Buoyancy due to air pockets and other components of internal buoyancy are instead assumed to continue to act on the structure as the water depth continues to rise. The same process of uplift load application and release is repeated for the other slabs in the structure when the inundation depth reaches and exceeds their elevation. The VDPO-BI analysis stops when the maximum lateral capacity of the structure under tsunami loads is reached.

**Figure 3:**
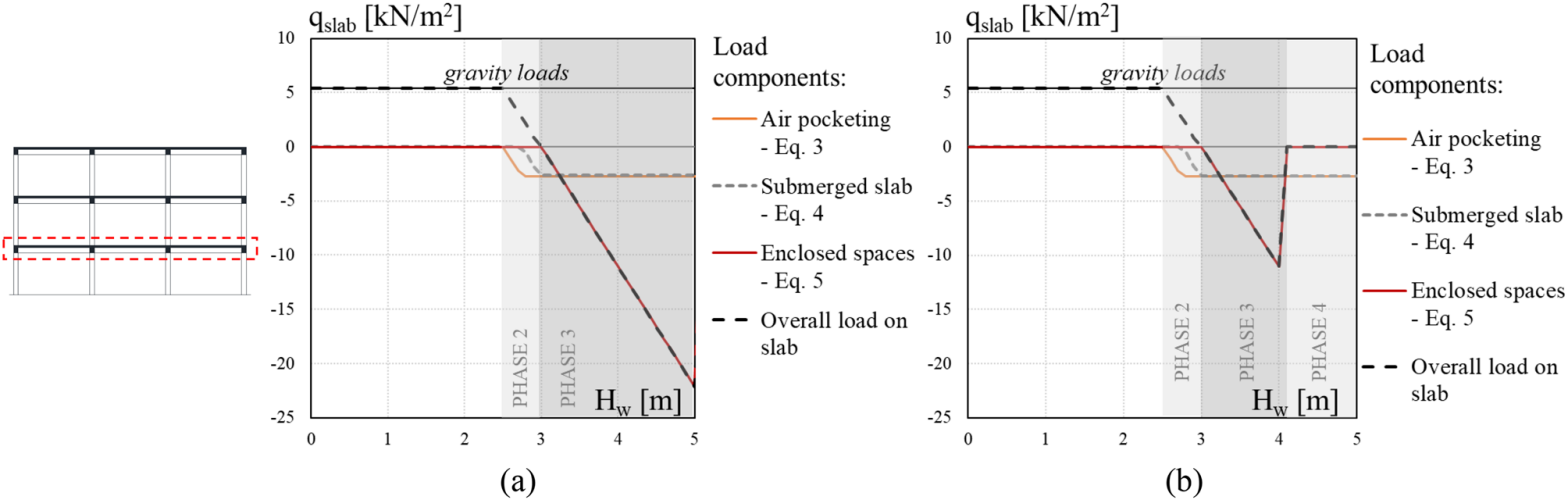
Tsunami buoyancy-induced uplift loads on slabs for (**a**) strong infill walls with high out-of-plane capacity (*H*_*OOP*_ = 2.5 m) and (**b**) weak infill walls with low out-of-plane capacity (*H*_*OOP*_ = 1.0 m).

The VDPO-BI analysis is force-controlled. The tsunami-induced lateral and uplift loads are applied on structural components as load time-histories, where each time step is associated to a value of tsunami inundation depth. To account for the failure of the infill wall and slabs, the value of inundation depth at which the capacity of infill walls and slabs are reached is first calculated, as discussed in "Characterization of uncertainties". The time histories for each structural node are then computed in advance of the VDPO-BI analysis, considering the damage condition of both the infill walls (see Fig. 2) and slabs for the inundation depth associated with each time step, so determining the appropriate area over which water pressures act. The magnitude and distribution of uplift loads on slabs change during the VDPO-BI analysis due to the development of the different components of internal buoyancy, as a function of the inundation depth and infill walls damage condition. To demonstrate the evolution of buoyancy-induced loads during a tsunami inundation, the uplift loads generated on the first story slab for a generic frame are shown in Fig. 3. For this preliminary application, gravity loads are assumed 5 kN/m^2^, the beam depth is assumed 500 mm, the slab thickness is 240 mm and the interstory height is assumed 3 m. The different uplift load components acting on the slab are plotted in Fig. 3a,b along with the overall vertical load for increasing inundation depth under the hypothesis of strong infill walls (high out-of-plane capacity, *H*_*OOP*_ = 2.5 m) and weak infill walls (low out-of-plane capacity *H*_*OOP*_ = 1.0 m), respectively. It is observed that even in the case of weak infill walls (which reduces the enclosed space component of uplift loads on the slabs), the buoyancy-induced uplift loads are larger than the gravity loads (i.e., higher than 5 kN/m^2^ but in the opposite direction in the example of Fig. 3). This means that high negative bending moments are induced at the midspan of slabs. Slabs that are not designed to sustain such kind of solicitation may fail. Such slabs are termed blow-out slabs in this paper. The uplift failure of slabs is explicitly considered in the VDPO-BI analysis, see Fig. 2d. Indeed, if the buoyancy-induced uplift loads overcome the capacity of the elevated slab during the incremental analysis, it is assumed that the slab has failed in all bays of the structure at that story level. In this case, vertical loads (both gravity and uplift loads) are no longer applied to that floor slab during the VDPO-BI. More details on the calculation of slab uplift failure are provided in Characterization of uncertainties.

**Figure 4:**
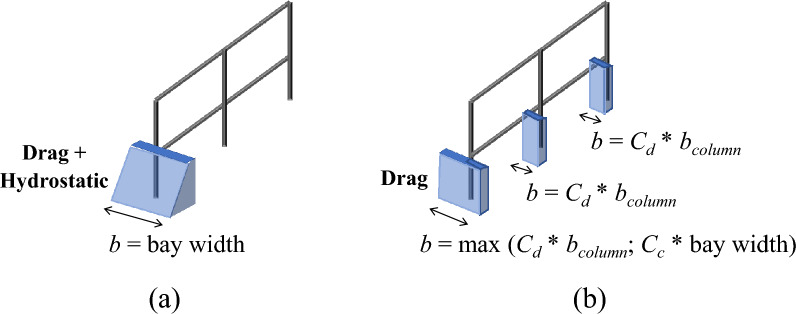
Impacting surface for tsunami-induced lateral loads accounting for drag amplification and damming debris accumulation: (**a**) undamaged exterior infill walls and (**b**) exterior infill walls broken away.

Tsunami-induced lateral loads are applied on structural and non-structural components in proportion to their impacting surface width, *b*. If the exterior infill walls are undamaged, *b* is equal to the infill length (i.e., bay width) for central columns and half infill length for corner columns. In such a case, lateral loads (i.e., unbalanced hydrostatic and drag) are applied to seaward structural components only, as depicted in Fig. 4a, assuming a perfect shear connection between RC frame and infill walls. Conversely, if the exterior infill walls in the plane normal to the tsunami direction are failed, lateral loads (i.e., drag) are applied to both exterior and interior structural components and should account for the effects of drag amplification and damming debris accumulation.

According to ASCE 7-22, hydrodynamic loads acting on single structural components should be amplified by a drag coefficient, *C*_*d*_, that depends on the geometry of the components and the closure ratio. Following the ASCE 7-22, *C*_*d*_ is assumed equal to 2 for rectangular columns in buildings with a closure ratio less than 0.2. Higher drag coefficients need to be used for structures with greater closure ratios to account for the increase in flow velocity inside the building. Hence, after the failure of breakaway infill walls, the impacting surface width *b* will be equal to the column width amplified by the drag coefficient for both exterior and interior columns. It is assumed that partitions, given their lower thickness and out-of-plane capacity with respect to exterior infill walls, suddenly fail when the exterior walls fail and the water enters the building. The width of the column (*b*_*column*_) may also comprise any residual portion of masonry walls that remain attached to the frame, depending on the type of connection between masonry and surrounding columns for the specific structure. The contribution of residual portion of masonry attached to frame should be treated as an uncertainty and is neglected in this preliminary analysis.

Damming debris are described in the ASCE 7-22 as waterborne debris that may accumulate on the front of the building after the failure of breakaway infill walls, increasing the impacting surface for seaward structural components. It is not clear if this damming debris accumulation should be considered only for the first storey level, where major debris accumulation is expected, or for the entire height of the building. To account for the effect of damming debris accumulation in the performance and fragility assessment of buildings with the VDPO-BI, the impacting surface width *b* for seaward components is computed as the bay width multiplied by a factor *C*_*c*_, representing the closure ratio (Fig. 4b). The minimum closure ratio allowed by the ASCE 7-22 for the design of tsunami-evacuation buildings accounting for the effect of openings and damming debris is 0.7. This closure ratio can be reduced to 0.5 for the case of open structures^4^. However, debris accumulation is highly aleatory, and *C*_*c*_ should be treated as un uncertainty for the development of fragility curves, being the probability of damming debris generation a function of the environmental conditions at the site of the structure and of the tsunami flow characteristics.

## Performance and fragility curves of case-study frames

To assess the significance of buoyancy-induced loads on structural response under tsunami inundation, the generalized VDPO-BI is applied in the performance and fragility assessment of three case-study frames representative of existing residential buildings in Mediterranean region. A damage scale is adopted for the performance assessment that considers local and global damage mechanisms. Furthermore, fragility curves are derived for the case study buildings, considering uncertainty in demand and capacity parameters. The tsunami fragility assessment is conducted under three scenarios with different assumptions on uplift loads and slabs failure, as summarized in Table 1. Details about the definition of case-study frames, finite element modelling, damage levels and uncertainties selected for this study are discussed in the following.

**Table 1:** Scenarios for performance and fragility assessment.

	Uplift loads	Blow-out slabs	Breakaway infill walls
*Scenario i*	✗	✗	✓
*Scenario ii*	✓	✗	✓
*Scenario iii*	✓	✓	✓

### Case-study frames

**Figure 5:**
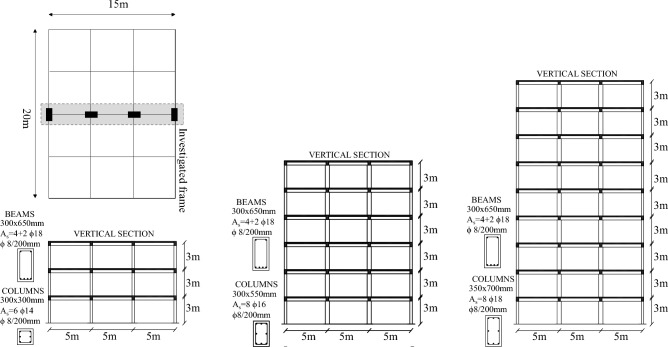
Case-study frames.

The case-study consist of three RC frames with different number of stories representing the central frames of a 3D residential building with unreinforced masonry infill walls. The buildings are defined with a simulated designed process following the Italian Royal Decree n. 2239^20^, to be representative of typical existing RC structures built before the 1980s in the Mediterranean area. The selected frames represent typical low-rise (i.e., 3 story), mid-rise (i.e., 6 story) and high-rise (i.e., 9 story) existing structures. The buildings are regular in plan and elevation, and Fig. 5 summarizes building plan data along with frames relevant structural details. Concrete with characteristic compressive strength *f*_*ck*_ = 20 MPa and reinforcement of characteristic yield stress *f*_*yk*_ = 380 MPa are used for design. Further information about the design procedure for case-study frames is reported in Del Zoppo et al.^8^. A concrete cover of 20 mm is adopted. The transverse reinforcement was not regulated in old design codes, and the stirrup spacing observed in gravity-design buildings before ‘80 s typically ranges between 200 and 250 mm. For the case-study frames, 8 mm bars diameter spaced at 200 mm are selected as transverse reinforcement for columns and beams based on typical construction practice, ensuring that the structural behaviour of such components under static and seismic conditions is governed by flexure.

**Figure 6:**
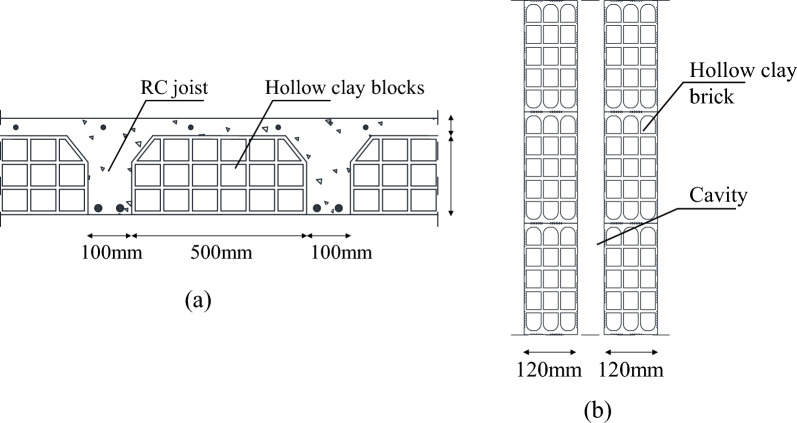
Details of typical slabs (**a**) and masonry infill walls (**b**) for existing RC buildings in Mediterranean area.

The interior slab typology typical of such existing structures consists in a cast-in-place RC ribbed slab, with constant joists dimensions (height 20 cm, width 10 cm and spaced 50 cm) and with a cover concrete layer of 4 cm thick. Among the joists, hollow clay masonry blocks having a lightening function for the slab are assumed and commonly used in the Mediterranean area, see Fig. 6a.

Typical masonry infill walls consist of two hollow clay brick masonry leaves (thickness 120 mm) with an internal cavity for thermal insulation, see Fig. 6b.

### Finite element modelling

The case-study frames are modelled as 2D frames, and the VDPO-BI non-linear static analysis is performed using the *OpenSees* software^21^. The RC frames are modelled using force-based nonlinear beam-column elements (i.e., forceBeamColumn elements) assuming five Gauss–Lobatto integration points^22^. Fibre sections are generated using the Patch command for the concrete, using a mesh size of 3 × 10 fibres for the cover elements and 10 × 10 fibres for the core. The steel reinforcement is modelled with the Layer command. Geometric nonlinearities, such as P-Delta effects, are considered in the finite element model. Beam-column joints are modelled by joining concurrent nodes, and no rigid links are adopted.

The axial load variation in vertical members caused by buoyancy induces a significant reduction in columns shear capacity at each step of the analysis. This variation in shear capacity as a function of the axial load acting on the columns cannot be updated step by step during the analysis. Hence, the shear failure of members is herein evaluated on analysis results employing the capacity model suggested by the Eurocode 8 part 3 for existing RC members^23^. This is to account for the effective axial load acting on structural members at each step of the analysis and to compute the actual shear capacity of columns reduced by the effect of buoyancy loads.

The mechanical behaviour of concrete in compression is modelled with the *Concrete04* material, representing the uniaxial Popovics^24^ material with degraded linear unloading/reloading stiffness^25^. Given the lack of transverse reinforcement and the low axial load in columns, the confinement effect of the core concrete is herein neglected. A bilinear stress–strain envelope, *Steel02* in *OpenSees*^26^, is adopted for the steel longitudinal reinforcement according to the uniaxial Giuffre–Menegotto–Pinto model with a smooth transition from the elastic to the plastic range.

A fixed restraint is adopted for the base of ground story columns. The RC slabs usually play a role of diaphragm for structures, redistributing external lateral forces to structural components. In the case of tsunami loading, where the water pressure is applied directly on vertical components, the role of diaphragm on the structural performance under tsunami loads can be compromised by the blow-out failure of elevated slabs. Numerical analyses have been conducted on models w/o diaphragm, and it has been observed that absence of the diaphragm constraint slightly affects the lateral performance of the frame. For this reason, the sudden loss of diaphragm effect caused by the uplift failure of blow-out slabs during the analysis is herein neglected and the diaphragm constraint is applied at each slab level in the models even for low-out slabs.

The non-structural components (i.e., masonry infill walls) are not included in the structural model, but their condition (undamaged or blown out) is considered during the preliminary definition of tsunami load time-histories for beams and columns to perform the VDPO-BI, as previously discussed in "Finite element modelling". Structural components are discretized in five nodes and load time-histories are assigned to each node of the structure. The out-of-plane capacity of masonry infill walls, *F*_*OOP*_, and the uplift capacity of the slab,* q*_*up*_, are computed as discussed in Sect. 4.5. These values are used as thresholds during the generation of tsunami load time-histories. In detail, tsunami loads are preliminary computed for increasing inundation depth assuming undamaged infill walls, as shown in Fig. 4a. If the *F*_*OOP*_ is reached, the infill walls are considered broken away and the distribution and magnitude of loads on columns are updated to account for the smaller area over which water pressures act, as reported in Fig. 4b. It should be noted that an equivalent uniform tsunami lateral pressure applied over the entire surface of the infill panel is computed to assess the out-of-plane failure of infill walls for increasing inundation depths. This is because the model adopted to compute the out-of-plane capacity of masonry walls is formulated for the case of uniform pressure distributed over the full height of the wall. The equivalent tsunami pressure is derived step-wise assuming an external work equal to that of the actual pressure distribution, as the infill wall experiences virtual out-of-plane displacements according to the double-arch failure mechanism^27^. Uplift loads on elevated slabs are also computed at each step of increasing inundation depth, and the overall uplift load is compared against *q*_*up*_. When the uplift capacity of the slab is reached, both uplift and gravity loads change to null values in the load time-histories applied to the beams to simulate the failure of blow-out slabs.

The in-plane behaviour of partitions is not herein considered due to the high variability of opening ratios observed in such walls in typical residential buildings, which can compromise the activation of struts. Dead loads are assumed to be 4 kN/m^2^ and live loads are 1.2 kN/m^2^. The Newton–Raphson algorithm is adopted to solve the nonlinear equations.

### Damage scale for performance evaluation under tsunami drag and buoyancy loads

The HAZUS Tsunami Model Technical Guidance^17^ provides international guidelines for the definition of damage levels for buildings subjected to tsunami inundation. HAZUS damage parameters are derived under the assumption of impermeable structures (i.e., with non-breakaway exterior walls) and neglect the local failure of structural components (i.e., shear failure of columns). Slight damage is not defined for tsunami conditions, and only Moderate, Extensive and Complete damage are considered, and are defined based on useability and economic loss considerations. Damage levels associated to structural damage are expressed in terms of lateral force (i.e., base-shear), with the Complete damage level being associated with the achievement of the peak lateral capacity for the structure. The Moderate damage level is associated with structural yielding, and Extensive damage is defined to occur at a base shear halfway between those for Moderate and Complete damage.

Starting from the HAZUS damage classification, mechanical structural damage levels are herein defined as summarized in Table 2. Damage levels are defined based on considerations about the functionality and useability of the structure in the aftermath of a tsunami, along with potential direct and indirect loss. Local damage to both vertical members (i.e., columns) and horizonal members (i.e., slabs) are also considered. Four structural damage levels are defined, from Slight damage to Complete (i.e., building unusable), in accordance with the HAZUS approach. The ductile mechanism is associated with the development of a flexural behaviour of structural members up to the achievement of the peak base shear (i.e., global numerical instability). This usually consisting in a soft-story mechanism, that compromise the building functionality and recovery.

**Table 2:** Damage scale and EDP threshold values.

	Slight	Moderate	Extensive	Complete
Structural damage definition	First achievement in any vertical member of concrete cracking (*M*_*cr*_)	First achievement in any vertical member of ½ steel yield strain (½ε_y_) in the longitudinal steel Or First achievement of slab flexural capacity (*q*_*up*_)	First achievement in any vertical member of steel yield strain (ε_y_) in the longitudinal steel Or First achievement in any vertical member of shear capacity (*V*_*R*_)	Peak base shear (*V*_*b*_) Or Achievement of the shear capacity (*V*_*R*_) in two consecutive vertical members

The extensive damage is associated with the first structural yielding, while a moderate damage level is considered to be reached at one-half of the yielding strain in one column. A slight damage level is introduced to include the concrete first cracking in RC columns.

Damage mechanisms related with the development of local brittle mechanisms are also herein considered. An extensive damage level is associated to the first shear failure in first story level columns. Indeed, numerical analyses attested the high probability of occurrence of shear failure in seaward columns^7,28^. Interior columns are less solicitated by tsunami-induced lateral loads and, hence, brittle failures are unlikely to occur in those members. A complete damage level is achieved when at least two consecutive seaward columns achieve their shear capacity, as this may represent the beginning of the loss of gravity load bearing capacity under the long duration lateral loads induced by the tsunami flow, and can compromise the stability of the entire building. Uplift failure of slabs is also explicitly considered as a damage mechanism. Although the failure of blow-out slabs does not compromise the overall structural stability of a building against gravity loads (i.e., not inducing any partial or total structural collapse), this local damage can compromise the functionality of an entire floor in the aftermath of a tsunami, with consequences on direct and indirect costs. Hence, the local damage mechanism of interior slabs at one storey is classified herein as a moderate damage level for the building, implying that the building is able to withstand gravity loads. Given the uniform distribution of uplift loads on slabs, the punching shear failure of slabs is not herein considered as a possible damage mechanism under tsunami-induced uplift loads.

The performance and fragility of the 2D case-study frames against tsunami-induced lateral and vertical loads is assessed through the VDPO-BI analysis as presented in "Finite element modelling". Fragility curves are produced for each structural damage mechanism identified in Table 2. It should be noted that the achievement of the peak base shear (i.e., complete damage) is computed neglecting the local shear failure of columns due to the modelling shortcomings previously discussed. However, this does not affect the considerations made in the following about the effect of buoyancy loads on fragility curves.

Local and global engineering demand parameters (EDP) are selected to monitor the structural response. Local EDPs consist in longitudinal steel strains and bending moments at critical sections of first storey level columns (i.e., top and bottom cross-sections), and shear forces acting on the first storey level columns. Overall uplift loads acting on elevated slabs are also considered as EDP for the damage mechanism associated to the blow-out slabs. The base shear (*F*) is assumed as global EDP to evaluate the structural capacity at complete damage. EDP thresholds for the achievement of each damage level (i.e., EDP_DL_) are also summarized in Table 2 (i.e., *M*_*cr*_ is the cracking bending moment, ε_y_ is the steel yielding strain). The columns shear capacity, *V*_*R*_, is computed according to EC8-3 provisions for existing structures as a function of the actual axial load. The flexural capacity of slabs against uplift loads strictly depends on the slab typology considered. Details on slabs capacity estimation are reported in "Characterization of uncertainties" for the case-study frames selected for this study.

### Tsunami fragility including buoyancy loads

Tsunami fragility assessment is performed through numerical analyses to assess the effect of buoyancy induced loads on the probability to exceed a certain structural damage mechanism, defined in Table 2, as a function of the tsunami intensity. The tsunami inundation depth, *H*_*w*_, is here adopted as a scalar intensity measure (IM) for the tsunami hazard, even though several studies identified other scalar and vector-valued tsunami parameters suitable to determine fragility curves^29,30^. However, the water depth is commonly the most adopted IM for empirical tsunami fragility functions, being easily monitored during post-tsunami in-situ inspections. Furthermore, given the relation between flow depth, Froude number and flow velocity (i.e., $Fr= u/\sqrt{{gH}_{w}}$, with *Fr* kept constant during the VDPO-BI), the scalar intensity measure *H*_*w*_ can be easily transformed in a vector-valued intensity measure IM = [*H*_*w*_, *u*]. The fragility curves are then modelled as a cumulative lognormal distribution of observed data from numerical analyses, with mean (*μ*_*DS*_) and logarithmic standard deviation (*β*_*DS*_) computed as mean and standard deviation of the actual distribution of scalar IMs.

### Characterization of uncertainties

In the probabilistic framework adopted to develop single-building tsunami fragility curves, uncertainties are taken into account for both capacity and demand parameters. It should be noted that the scope of the paper is not to provide fragility functions for RC buildings, but instead to address the effects of modelling buoyancy loads and blow-out slabs on the fragility of frames. Uncertainties about the geometry of case-study buildings are neglected in the single-building fragility assessment. Uncertainties on capacity are related to material properties to be adopted for structural and non-structural components, as well as capacity models to be used to assess threshold values for the infill walls out-of-plane capacity and the slabs uplift failure. Conversely, uncertainties on tsunami demand are mainly related to the Froude number characterizing the tsunami flow at the building location. Uncertainties on demand also involve the load combination to be used during the performance assessment. According to ASCE 7-22 provisions, two load combinations should be considered for tsunami design, where *D* are dead loads, *L* are live loads, and *T*_*su*_ are tsunami lateral and buoyancy loads:

6 $$  \begin{gathered}   COMBO\;1)\;0.9{\text{D}} + {\text{Tsu}} \hfill \\   COMBO\;2)\;1.2{\text{D}} + 0.5{\text{L}} + {\text{Tsu}} \hfill \\  \end{gathered}  $$

Uncertain parameters are considered as independent random variables. The Latin Hypercube algorithm and Monte Carlo simulation are adopted to generate a sample of 10^3^ random combinations of the selected variables, each corresponding to a building possible realization.

The fragility function for a specific damage level is then computed on the sample of 10^3^ random combinations as *P* [IM < IM|EDP = EDP_DL_].

A set of aleatory variables with normal or uniform distribution are defined to explicitly account for uncertainties related to capacity and external demand, as summarized in Table 3. For variables with a uniform distribution, the range of variability is reported; conversely, in the case of normally distributed variables, both mean and *CoV* (in brackets) are indicated.

**Table 3:** Uncertainties for fragility assessment of case-study frames.

Variable	Distribution	Mean (CoV) or range of variation
Capacity		
* f*_*s*_	Normal	570 MPa (10%)
* f*_*c*_	Normal	22 MPa (10%)
* F*_*OOP*_	Normal	26.3 kN (53%)
* q*_*up*_	Normal	5.5 kN/m^2^ (11%)
Demand		
* Fr*	Uniform	0.7–2.0
* Load combination*	Uniform	*COMBO 1* or *COMBO 2*

There is little consensus about the appropriate characterization of uncertainties related to material properties in existing RC buildings. The present study adopts statistics from relevant studies performed in Mediterranean regions that derived fragility functions of existing buildings and building stocks^31,32^. Mean values of mechanical properties are considered for the performance assessment of the RC frames. To obtain mean values, the concrete characteristic compressive strength is multiplied by a factor *γ*_*c*_ = 1.1, and the steel strength is multiplied by *γ*_*s*_ = 1.5^33^. To account for uncertainties in steel and concrete mechanical properties for the single-building fragility assessment, yielding and compressive strength are considered as normally distributed random variables with a *CoV* = 10%^31^. Statistics for the out-of-plane capacity of typical exterior infill walls, *F*_*OOP*_, are derived using the double-arch analytical model reported in Del Zoppo et al.^8^ through Monte Carlo simulation on 10^3^ samples, assuming as random variables the masonry mean compressive strength of 1.5 MPa (*CoV* 14%) and the masonry elastic modulus of 1.8 GPa (*CoV* 14%)^32^. Given the internal cavity of the typical infill walls configuration considered for the case-study frames, only the thickness of the outer layer of bricks is considered for computing the infill walls capacity (i.e., 120 mm). This implies a very weak capacity of infill walls in the out-of-plane direction.

Uncertainties on the uplift capacity of slabs, *q*_*up*_, are also considered within the fragility assessment framework. The flexural capacity of the RC slabs is conservatively computed using sectional analysis as the flexural capacity at first cracking, *M*_*cracking,slab*_, given the lack of tensile reinforcement at the mid-span under negative bending moment:

7 $$ M_{cracking,slab} = f_{t} I/y_{G} $$

where *f*_*t*_ is the concrete tensile strength, computed as 0.3*f*_*c*_^2/3^ as per Eurocode 2—Part 1^34^, with *f*_*c*_ the concrete compressive strength, *I* the inertia of the slab cross-section and *y*_*G*_ the distance of the upper concrete fibre from the centroid of the section. The uplift load capacity is then calculated from:

8 $$ q_{up} = kM_{cracking,slab} /L^{2} $$

where *L* is the span of the slab (i.e., equal to 5 m) and *k* a coefficient depending on bending moment diagram. Statistics for *q*_*up*_ are computed running a Monte Carlo simulation on 10^3^ samples, assuming a mean concrete compressive strength *f*_*c*_ of 22 MPa (*CoV* 10%) and *k* ranging between 9 and 13, assuming a uniform distribution.

Tsunami lateral loads are computed assuming a dense urban environment, *B/w* = 0.6 (i.e., *C*_*D*_ = 4.7, *λ*_*s*_ = 2.0, *Fr_c* = 0.32), and a Froude number ranging between 0.7 and 2.0 for this application. Although debris accumulation should be considered for the tsunami fragility assessment, for the present study, a negligible damming debris accumulation at the site of the structure is assumed (i.e., *C*_*c*_ = 0). This is deemed acceptable as the focus of this paper is to assess the influence of vertical loads on structural performance, and significant lateral loads are already considered to be attracted to the structure due to the presence of infill walls. Given this assumption, the case study buildings can be considered as representative of buildings directly facing the shoreline, and away from significant sources of damming debris. It should be noted that such structures may also be exposed to the impact loads from floating debris (i.e., boats)^1^.

Both load combinations in Eq. (6) are herein randomly considered to combine gravity and tsunami-induced loads to develop fragility curves.

## Results

The effects of buoyancy-induced loads and blow-out slabs on the damage mechanisms and associated fragility curves of the three case-study frames are herein illustrated and discussed. Considerations are also made relatively to the consequent effect on tsunami vulnerability and loss estimation.

### Effect of buoyancy loads on damage mechanisms

Selected damage mechanisms occurrence is illustrated for the case-study frames under the three scenarios reported in Table 1: (scenario *i*) uplift loads on slabs are neglected during the analysis; (scenario *ii*) uplift loads are considered but the uplift failure of the slab is neglected (i.e., non-blow-out slabs); (scenario *iii*) uplift loads and blow-out slabs are both considered. The case-study frames are analysed assuming mean values for the random variables related to the capacity. The analyses are performed assuming the load combination 1 (Eq. 6) and a Froude number of 0.7, which is the lower bound of the selected range of investigation (Table 3). For the considered configurations, the failure of breakaway infill walls is reached for an inundation depth of 0.8 m at first storey level and of 3.8 m at second storey level.

**Figure 7:**
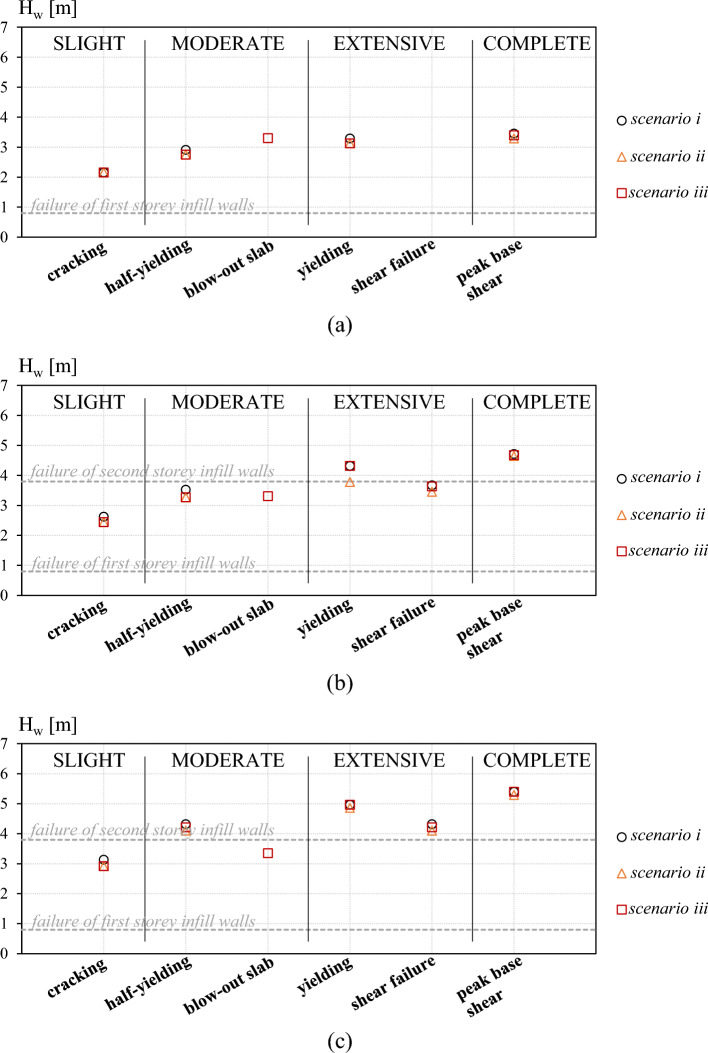
Damage mechanisms for a Froude number of 0.7: low (**a**), mid (**b**) and high-rise (**c**) case-study frames.

Figure 7 shows the damage mechanisms occurrence for the three case-study frames as a function of the inundation depth. The damage mechanism associated with the blow-out slab failure is computed only for scenario *iii*, where uplift loads and blow-out slabs are considered. In should be noted that, before the blow-out failure of slabs, scenario *ii* and scenario *iii* provide same results. The Complete damage associated with the shear failure of two consecutive columns is never achieved in the 2D case-study frames, as only seaward columns reach such a brittle failure mechanism.

The plots show that uplift loads affect the occurrence and evolution of damage mechanisms for the three frames. Indeed, the inclusion of uplift loads in the analysis anticipates the occurrence of EDP thresholds for the considered damage mechanisms, especially if non-blow-out slabs are assumed (scenario *ii*). The blow-out slab failure is reached for an inundation depth of 3.3 m for all the frames, having same geometrical and mechanical properties and same load conditions. For the low-rise frame (Fig. 7a), all damage mechanisms in scenarios *ii* and *iii* reached before this value of H_w_ are affected by buoyancy (i.e., moderate to complete). For the specific configuration, buoyancy loads reduce by 5% the inundation depth for which the damage mechanisms are reached. For the mid-rise frame, the effect of buoyancy loads is more visible on the Slight to extensive damage mechanisms (Fig. 7b). In detail, in scenario *ii* damage mechanisms occur for inundation depth values 6–14% lower than in scenario *i* where uplift loads are neglected. The most evident difference among different scenarios is related to the ductile Extensive damage, where the out-of-plane failure of the second storey infill walls play a fundamental role in the damage evolution. Indeed, the failure of the exterior cladding induces a significant reduction of uplift loads on the slab that allows the achievement of higher inundation depth levels in scenarios *i* and *iii* with respect to scenario *ii* where the damage mechanism is reached before the infill walls failure. Conversely, the complete damage is reached for all scenarios after the failure of second storey infill walls and, for this reason is only slightly affected by the uplift loads acting on first storey slab (i.e., only due to air pockets and submerged slabs). Similarly, for the high-rise frame the damage level more affected by uplift loads is the one reached before the failure of second storey breakaway walls (i.e., Slight damage, inundation depth 7% lower than in scenario *i*). Other damage levels are only slightly affected by the uplift loads.

It is observed that blow-out slabs, when considered (scenario *iii*), anticipate the achievement of a Moderate damage level for the mid and high-rise frames, see Fig. 7b,c. After the failure of blow-out slabs, the damage evolution for scenario *i* and *iii* is the same for the three frames. Furthermore, the failure of fist storey slabs is achieved for all frames before the Collapse. The uplift failure of slabs and the out-of-plane failure of infill walls delay the damage mechanisms associated with vertical structural members. Indeed, they allow for a reduction of tensile axial loads on columns due to buoyancy (see Fig. 3b) and, hence, for a greater flexural and shear capacity.

It is also observed for the investigated configuration that the local shear failure of vertical members is not reached for the low-rise frame, whereas it is the first Extensive damage mechanism occurred in the mid and high-rise frames.

It is worth noting that the observations made herein about the damage mechanisms occurrence and evolution are significantly affected by the selection of the Froude number (i.e., Fr = 0.7), as further discussed in "Effect of buoyancy loads on tsunami fragility curves".

### Effect of buoyancy loads on tsunami fragility curves

**Figure 8:**
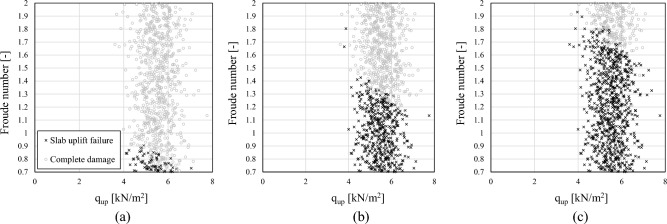
Occurrence of first story slab uplift failure for low (**a**), mid (**b**) and high-rise (**c**) case-study frames.

A probabilistic analysis is conducted to include uncertainties for assessing the probability of occurrence of damage mechanisms previously investigated and point out the effect of buoyancy loads on structural fragility. It should be noted that the EDP threshold associated with damage to horizontal members (i.e., blow-out slabs) is not always reached during the VDPO-BI analysis. Indeed, in several cases the global numerical instability of the model is reached before the uplift failure of the slab occurs. The rate of occurrence of slab uplift failure with respect to complete damage is shown in Fig. 8 for the sample of case-study frames as a function of the Froude number and the uplift capacity of the slab (q_up_). For the low-rise frame, slab uplift failure is achieved before the complete damage in only 7.5% of investigated cases, see Fig. 8a. This strongly depends on the magnitude of tsunami lateral load, expressed by means of the Froude number, rather than from the slab capacity. Indeed, for Froude number greater than 0.9 the complete damage is always achieved before the slab failure. A higher rate of occurrence of slabs uplift failure is observed for mid (46.5%) and high-rise frames (77.7%), given the greater dimension of columns at the first story level which provides a larger lateral capacity with respect to the low-rise frame. For such case-study frames, the tsunami Froude number governs the occurrence of interior slabs uplift failures, as shown in Fig. 8b,c. Hence, fragility curves associated to the occurrence of blow-out slabs are not computed for the full sample but only for those realizations in the sample where the uplift failure of slab occurred before the global numerical instability. It should be noted that this result is also affected by the out-of-plane resistance of infill walls. Indeed, strong infill walls may induce a higher probability of failure of interior slabs before the Complete damage of the structure with respect to the case in weak infill walls herein investigated.

**Figure 9:**
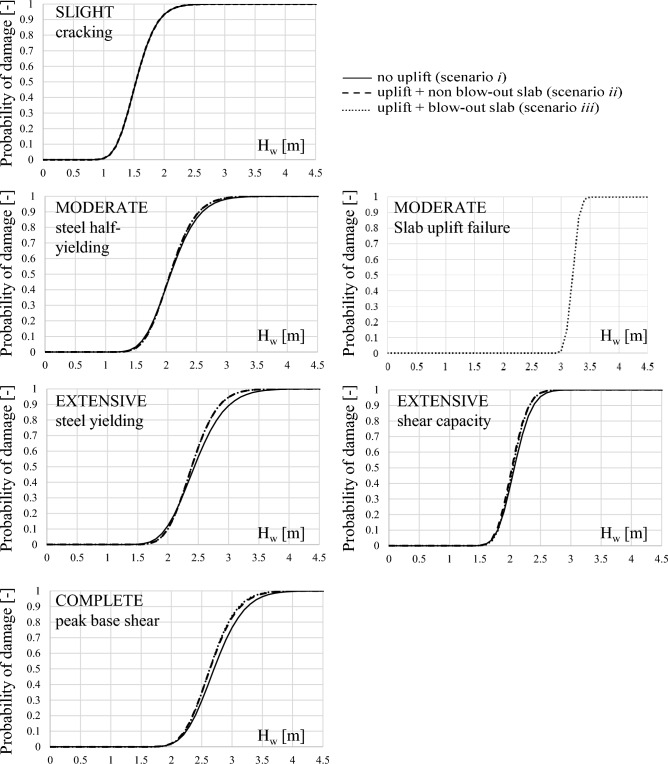
Effect of buoyancy loads on fragility curves for damage mechanisms on the low-rise case-study frame.

The effect of buoyancy loads on fragility curves is assessed and presented separately for each damage mechanism. Fragility curves derived for the low-rise frame are first compared in Fig. 9 for the three investigated scenarios. The Figure shows that buoyancy-induced uplift loads only slightly affect the damage mechanisms associated with the concrete cracking and the ½ steel yield strain. This is because the inundation depth associated with these damage levels is usually lower than the soffit of first story slab, and no significant uplift loads are acting on the elevated slabs when the damage occurs. Conversely, damage mechanisms associated with steel yield strain, shear capacity of columns and peak base shear usually occur when tsunami uplift loads are already acting on the slabs due to buoyancy. Indeed, the fragility associated to these damage mechanisms for the low-rise frame under scenario *ii* and *iii* is slightly increased (i.e., reduction in median and logarithmic standard deviation), with respect to scenario *i* due to the effect of uplift loads on non-blow-out slabs. For instance, the probability of occurrence of the steel yielding (i.e., Extensive damage) for the low-rise frame for H_w_ = 2.6 m is 65% in scenario *i* and 72% in scenarios *ii* and *iii*. Similarly, the probability of occurrence of Complete damage for H_w_ = 3.0 m is 39% for scenario *i* and 46% for scenarios *ii* and *iii*. It should be noted that the difference in fragility curves between scenarios *ii* and *iii* is almost negligible for the low-rise frame, given the low rate of occurrence of slabs uplift failure (i.e., 7.5%).

**Figure 10:**
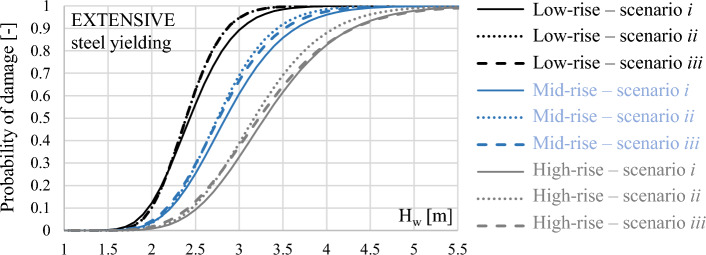
Comparison of fragility curves at Extensive damage level for low, mid and high-rise frames.

Conversely, for the mid and high-rise frame, fragility curves for the Extensive to Complete structural damage under scenario *iii* are slightly different with respect to scenario *ii* due to the achievement of the slabs uplift failure, and hence they are more similar to fragility curves of scenario *i*. Figure 10 shows a comparison of fragility curves associated with the steel yielding (i.e., Extensive damage) for the three case-study frames under different scenarios. The plot attests the increase in fragility for the three frames under scenario *ii*, where blow-out slabs failure is neglected, and shows the effect of blow-out slabs consideration on fragility curves in scenario *iii*. Indeed, it observed that the curves in scenario *iii* progressively move from scenario *ii* to scenario *i* with the increasing height of the building. The general observations derived for the low-rise frame about the effect of uplift loads on fragility curves are also valid for the mid and high-rise frames, and data are not herein reported for the sake of brevity. Differently from the case of low-rise frame, for the mid and high-rise frame the fragility curves related to the achievement of ½ steel yield strain are affected by the effect of uplift loads. Indeed, for instance for an inundation depth H_w_ = 3.0 m the probability of damage for the mid-rise frame is 86% in scenario *i* and 93% in scenarios *ii* and *iii*. Similarly, for the high-rise frame the probability of damage is 64%, 73% and 71% respectively for scenario *i*, *ii* and *iii*. Overall, it is observed that the inclusion of buoyancy loads increases the probability of occurrence of damage mechanisms up to 20% with respect to the case where buoyancy is neglected. Median and logarithmic standard deviation defining the derived fragility curves are summarised in Table 4 for all frames and scenarios.

**Table 4:** Fragility curves: median (μ) and logarithmic standard deviation (σ).

	μ (m)	Σ	μ (m)	σ	μ (m)	σ	μ (m)	σ	μ (m)
Low-rise frame									
Scenario *i*	0.43	0.18	0.73	0.18	0.89	0.17	0.99	0.14	0.73
Scenario *ii*	0.43	0.18	0.72	0.16	0.87	0.14	0.97	0.13	0.71
Scenario *iii*	0.43	0.18	0.72	0.16	0.87	0.14	0.97	0.14	0.71
Mid-rise frame									
Scenario *i*	0.57	0.18	0.88	0.20	1.05	0.19	1.18	0.15	0.79
Scenario *ii*	0.57	0.18	0.86	0.16	1.01	0.17	1.16	0.14	0.78
Scenario *iii*	0.57	0.18	0.86	0.16	1.02	0.19	1.17	0.16	0.78
High-rise									
Scenario *i*	0.72	0.18	1.02	0.21	1.19	0.21	1.31	0.18	0.90
Scenario *ii*	0.72	0.17	0.99	0.18	1.15	0.20	1.30	0.19	0.88
Scenario *iii*	0.72	0.17	0.99	0.19	1.17	0.23	1.31	0.19	0.88

Fragility curves associated with the damage to horizontal components (i.e., blow-out slabs) are quite steep, as shown in Fig. 9 for the low-rise frame. This is because the failure of the slab mainly depends on the slabs’ capacity and on the volume of enclosed spaces over the slab that generates the internal buoyancy (which is a function of the inundation depth and the out-of-plane capacity of infill walls). Indeed, the fragility curves associated with the slab uplift failure have very similar mean and logarithmic standard deviation for the low, mid and high-rise case-study frames.

Focusing on the Moderate damage level, it is observed that the probability of occurrence of slabs uplift failure is usually lower than the exceedance of the half-yielding of longitudinal steel bars in vertical members for the three case-study frames herein analysed. Indeed, for the low-rise frame the slab uplift failure is never achieved before the steel half yielding; conversely, for the mid and high-rise frames the rate of occurrence of slabs uplift failure before the half-yielding is 0.3% and 17.8%, respectively. Hence, for the case-study frames herein considered, the fragility curves associated to a Moderate damage level are mainly governed by the steel half-yielding.

## Discussion

The damage assessment of the three case-study frames representative of Mediterranean existing buildings attests that the inclusion of buoyancy loads during the performance analysis affect the occurrence of damage mechanisms, anticipating is some cases those associated to extensive and complete damage for structures with non blow-out slabs. Conversely, for structures with blow-out slabs, it is observed that the uplift failure of interior slab is a possible damage mechanism for existing RC buildings during a tsunami inundation, with a rate of occurrence up to 78% for the investigated frames. It is also found that the probability of occurrence of such damage mechanism increases with the number of storeys of the building, affecting both reparation costs and the usability of the structure as a shelter during a tsunami inundation. Indeed, costs related to the repair or replacement of the slabs should be accounted for in the evaluation of direct losses. Indirect losses should also account for the fact that uplift failure of interior slabs will make the building unusable in the aftermath of a tsunami, affecting downtime. As a result, in the case of loss or useability assessment it is very important that the probability of uplift failure of interior slabs (and hence buoyancy-induced loading) is explicitly computed, especially for mid to high rise structures. For loss assessment purposes, local structural analyses can also be conducted to assess the probability of uplift failure of interior slabs.

It is also observed that structures with blow-out slabs are less prone to a complete damage than those ones with non blow-out slabs. Thus, the adoption of properly designed breakaway holes in interior slabs, suggested in Robertson^18^ for the design on tsunami evacuation shelters, may also represent a suitable retrofit solution for existing buildings, allowing to reduce the uplift loads on the structure whilst better controlling damage (i.e., retaining a portion of the slab and limiting damage induced to other structural elements that might happen under a normal slab uplift failure). This, in addition to local strengthening solutions to avoid brittle failure mechanisms and enhance the flexural capacity and ductility of RC members^35–37^, among others, may reduce the vulnerability of existing RC frames to tsunami loading.

For the particular case-study buildings studied, it is observed that the consideration of uplift loads on slabs slightly affects fragility curves as compared to the condition where uplift loads and slabs uplift failure are neglected. For the case-study buildings analysed this small change (i.e., lower than 20%) seems not to justify the computational effort associated with the use of such kind of refined analysis for fragility assessment, especially at large scale. This finding would also suggest that analytical fragility curves produced so far for Mediterranean RC buildings ignoring buoyancy loads are not affected by a significant error due to this omission. It is worth noting that such conclusions are strictly related to the damage scale definition adopted in the study.

## Conclusions

The paper investigates the effect of internal buoyancy loads on the structural damage and fragility of buildings under tsunami loading. To this aim, a generalised structural analysis methodology for the performance assessment of frames with breakaway infill walls, termed VDPO-BI, able to integrate the tsunami-induced lateral and buoyancy loads on structures, and to account for blow-out slabs is adopted. The paper investigates the effects of modelling buoyancy-induced loads and blow-out slabs on the fragility of existing, non-seismically designed, masonry infilled RC frame buildings of different heights. The main findings and conclusions of the study are herein summarized:

- Tsunami-induced buoyancy loads significantly affected the structural damage of case-study buildings under tsunami inundation, inducing uplift failure of blow-out slabs especially in mid to high-rise frames;
- The rate of occurrence of blow-out slab failures due to uplift loads increases with the number of stories (i.e., from 7 to 78%) and reduces when the Froude number increases. However, the occurrence of uplift failure of slabs depends on several other independent factors, namely: the out-of-plane capacity of exterior infill walls, the depth of interior beams, the location of openings in the exterior infill above the slab (that determine water ingress and retention) and the capacity of the slab itself;
- The performance analysis conducted clearly demonstrates that buoyancy-induced loads play a key role in the structural damage evolution during the incremental analysis. Most published studies do not consider buoyancy loads at all, nor do they consider the capacities of slabs and infill walls. Such studies may result in unrealistic damage mechanism predictions, that can compromise the effectiveness of any strengthening strategies designed based on their results;
- In the presented single-building fragility curves, buoyancy-induced uplift loads on slabs are seen to slightly increase the fragility curves of the investigated case-study frames for the selected damage mechanisms with respect to the case where buoyancy loads are neglected during the VDPO-BI analysis. Neglecting buoyancy loads during the fragility analysis leads to underestimating the probability of damage up to 20% for the investigated frames;
- Neglecting buoyancy loads and blow-out slabs does not allow for a proper estimation of economic losses. Indeed, the uplift failure of interior slabs can cause significant direct and indirect losses associated with the serviceability of the building in the aftermath of a tsunami that need to be accounted for during a loss assessment.

It is important to note that the derived conclusions are strictly related to the type of infill walls considered. Indeed, infill walls with higher out-of-plane capacity than those considered in this study (i.e., new or retrofitted walls, for instance) may lead to high uplift loads effects on fragility curves. The presented conclusions promote the inclusion of buoyancy-loading in the tsunami damage assessment of buildings, and although they are based on a limited number of case study applications, they make engineering sense. Further case-study applications will be required to test the generality of the conclusions for other buildings and different tsunami inundation conditions.

## Data Availability

The datasets generated during the current study are available from the corresponding author upon reasonable request. Please contact the corresponding author: Dr Marta Del Zoppo (m.delzoppo@ucl.ac.uk).
